# Editorial: Cognitive-related and connectome-based biomarkers for depression: the application of state-of-the-art techniques and models to uncover

**DOI:** 10.3389/fpsyt.2023.1249049

**Published:** 2023-07-27

**Authors:** Zhiyi Chen, Zhengzhi Feng, Qin Dai, Sirois Fuschia

**Affiliations:** ^1^Experimental Research Center of Medical and Psychological Science, School of Psychology, Third Military Medical University, Chongqing, China; ^2^School of Psychology, Third Military Medical University, Chongqing, China; ^3^Department of Psychology, Durham University, Durham, United Kingdom

**Keywords:** depression, biomarker, connectome, cognitive component, neuroimaging

Depression, particularly major depressive disorder (MDD), is one of the key factors contributing to human disability and suicide, with almost 800,000 deaths for depression-related suicide per year (1). In addition, the economic burden of MDD are increasing faster than ever across the globe, especially in depression-related unemployment, homelessness, and diagnostic/therapeutic costs (2, 3). Thus, these issues necessitate the development and validation of low-cost, rapid, reliable, and interpretable biomarkers for MDD. To do so, many efforts had been made to probe into neuromarkers of MDD from neuroimaging-related studies with local neural features, which not only showed high measure reliability and neural interpretability but also provided tremendous insights into neuropathological substrates of this psychiatric condition (4). Despite these promising biomarkers, connectome-based neural architecture, in conjunction of state-of-the-art techniques and groundbreaking conceptional models, is increasingly becoming a promising direction to capture intrinsic brain pattern as novel neuromarkers in MDD diagnostics (5).

Traditional diagnostics based on structured interviews (i.e., the Diagnostic Statistical Manual, DSM-5) are somewhat grounded on subjective observations and judgement from clinicians (6). Thus, neuroimaging-aid diagnostics, especially in leveraging connectome-based features that could sensitively capture intrinsic brain patterns, may complement DSM-related ones to be more reliable (7). Further, building artificially intelligence (AI) to individually predict prognostic outcome for each MDD patient from such neuroimaging-based markers is under rapid developments in clinical research (8). In other words, capitalizing on neuroimaging-based neuromarkers, in conjunction of cutting-edge AI techniques, may pave the promising way for precision medicine on MDD patients in the near future. In total, the primary aim of this Research Topic is to enhance our understanding of cognitive-related and connectome-based biomarkers of MDD by drawing on state-of-the-art techniques or models.

To tackle the issue of the poor diagnostic performance of single neural/biological approaches, Zhang et al. capitalized on multi-modal components comprised of intrinsic spontaneous brain activities, eye-movement patterns, and event-related potentials for distinguishing MDD from healthy controls. By training machine-learning model, Zhang et al. found the increased fractional amplitude of low-frequency fluctuations (fALFF) in sensorimotor network and cerebellar areas could classify MDD patients at a plausible accuracy of 78.6%. A further interesting finding was that such features were significantly correlated with depressive symptoms. Thus, rather than a self-reported observation, this study not only revealed a robust neural marker for diagnosing MDD and even for predicting severity of clinical symptom, but also shed light on the potential brain functional anomalies of MDD. Overall, by taking advantage of the prominent advances in machine-learning models and multi-modal cognitive/neural/biological features, their work highlights a pathway to diagnose and prognose MDD using neural hallmarks.

In addition to neuroimaging features, dysfunctions in electrophysiological activities have been examined as promising candidates for diagnostic biomarkers in MDD (9). Consistent with this, Shi, Sun et al. conducted face perception tasks to investigate specific event-related potential (ERP) components for emotional processing in MDD. Importantly, their study further probed into how this process may be moderated by the aging effects. Their findings revealed that the N170 component in middle-aged MDD patients was statistically significantly larger than that of healthy-control peers. However, these findings were but not generalizable to younger adult patients. There were also parallel findings for the associations of N170 inversion/effect to MDD-specific symptom. Such aging effects were found to be marginal in the healthy controls, that was, neither the N170 component nor face perceptional effects (e.g., inversion effect) were distinct between young and middle-aged patients. In other words, N170 potential in early-stage perceptional process may be not a generalizable biomarker but an age-specific electrophysiological pattern in middle-aged MDD patients.

Beyond neural information, heart rate variability (HRV) from 24-h Electrocardiogram (ECG) is a cost-less and promising biomarker for prognosis across multiple medical specialties. HRV reflects the variation in time intervals between consecutive heartbeats that can be measured in terms of either time domain or frequency domain analyses (10). Bringmann et al. utilized HRV to predict the prognostic outcome of a Meditation-Based Lifestyle Modification (MBLM) treatment for outpatients with mild to moderate depressive symptoms. Both a minimal treatment group (MINMAL, drug-used only) and a treatment-as-usual group (TAU, best clinical treatment practice) were added as control conditions to test the effectiveness of the MBLM. Their findings demonstrated that HRV changes occurred from MBLM to TAU. Notably, not only were the Rényi entropy of symbolic dynamics and the vagal tones mediating RMSSD increased alongside the changes in HRV for MBLM-treated outpatients, but no changes were found in either the MINMAL group nor the TAU group. These findings support the conclusion that HRV may be a rapid and cost-less marker for identifying outpatients with depressive symptoms.

As noted above, one fundamental aim worth mentioning for the current Research Topic is to probe the complex biological/neural mechanisms of depressive disorders. To this end, Shi, Hu et al. substantially extended our understanding of depression-related neuromarkers, from macro-scale brain localizers to cellular neural circuits, by using a chronic social defeat stress mouse model. Their study provided support for a long-standing assumption about psychopathology by demonstrating an association between stress and depression-like behavior, as well as revealing the protective roles of social and sexual reward in moderating the aftermath of stress on depression. Another crucial finding was that the mRNA expression levels of CB1 and metabolic glutamate receptor 5 (mGluR5), the protein expression level of mGluR5, and the expression level of 2-AG (2-arachidonoylglycerol) in both ventral tegmental area (VTA) and dorsal raphe nucleus (DRN) were upregulated by reward in this stress-induced depression animal model. This study further provided causal evidence to support that CB1 over-expression could reduce depression-related behaviors. Taken together, the findings from Shi, Hu et al.'s well-designed animal-model experiment indicate that rewards may moderate stress resilience by regulating ECs and mGluR5 in VTA and DRN (all promising cellular biomarkers for depression) to induce depression-related behaviors.

Overall, we are all pleased and thankful for these contributions to the special issue as they provide compelling evidence that advances our understanding of how novel and up-to-date neural (i.e., fALFF and N170 potentials), biological (i.e., HRV), and even cellular hallmarks (i.e., mGluR5 and 2-AG) can aid in the diagnosis and prognosis of depression. Despite no direct primary studies relating to connectome-based or cognitive-related neuromarkers, these articles still provided novel candidates favoring to MDD diagnostics or prognostics. By creating this Research Topic we hope that state-of-the-art techniques and multi-modal biomarkers featured in the research can enhance our understanding of the biological mechanisms of depressive disorders, and contribute to of cutting-edge treatment models in psychiatric practice.

## Author contributions

All authors listed have made a substantial, direct, and intellectual contribution to the work and approved it for publication.
